# Oviposition-Induced Volatiles Affect Electrophysiological and Behavioral Responses of Egg Parasitoids

**DOI:** 10.3390/insects10120437

**Published:** 2019-12-05

**Authors:** Panagiotis G Milonas, Eirini Anastasaki, Georgios Partsinevelos

**Affiliations:** Laboratory of Biological Control, Department of Entomology & Agricultural Zoology, Benaki Phytopathological Institute, 8 S. Delta Street, 14561 Kifissia, Greece; e.anastasaki@bpi.gr (E.A.); g.partsinevelos@bpi.gr (G.P.)

**Keywords:** *Trichogramma*, tomato leafminer, olfactometer

## Abstract

In response to an attack by herbivores, plants emit a variety of compounds that may act as semiochemicals. Oviposition-induced volatiles (OIPVs) have been shown to mediate interactions between plants and natural enemies. Here, we investigated the role of OIPVs by *Tuta absoluta* towards two egg parasitoids, *Trichogramma cordubense* and *T. achaeae*. We collected headspace volatiles from tomato plants at 24, 48, and 72 h after oviposition by *T. absoluta* females and tested the antennographic response of *Trichogramma* parasitoids to them by means of gas chromatography- electro-antennographical detection (GC-EAD). The response of the parasitoids was also tested in behavioral experiments using a Y-tube olfactometer. Oviposition by *T. absoluta* females induced qualitative and quantitative changes in the volatiles emitted by tomato plants. Antennae of *Trichogramma* parasitoids responded to several of the induced volatiles in GC-EAD. *T. cordubense* females were attracted to tomato plants with *T. absoluta* eggs 24 h after oviposition. The elucidation of the behavior of egg parasitoids towards OIPVs enhances the development of sustainable management strategies either by selecting species that exploit OIPVs or by manipulating their foraging behavior by utilizing specific OIPVs that are used by parasitoids as a host location.

## 1. Introduction

Plants under attack by herbivorous insects produce semiochemicals. These may directly protect the plant either by their toxic properties or by being repellent to conspecific or heterospecific herbivorous species. Indirectly, they may attract natural enemies antagonistic to the herbivores [1,2,3,4,5,6]. The production of herbivore-induced plant volatiles (HIPVs) that act as foraging cues for parasitoids and predators is known to be triggered by the feeding activity of insects on host plants [1]. Recently, the oviposition of herbivorous insects alone or in combination with feeding has been proven to induce the emission of oviposition-induced volatiles (OIPVs) that act as synomones [7,8,9]. Plants benefit by responding to oviposition as they switch on defense mechanisms early before any damage occurs to the plant [10,11]. Several studies have shown that egg deposition alone induced the emission of OIPVs that attracted egg parasitoids that eventually kill their hosts [12,13,14,15,16].

The tomato leafminer, *Tuta absoluta* (Meyrick) (Lepidoptera: Gelechiidae), is a major pest of tomato, *Solanum lycopersicon* L. (Solanacae), throughout South and Central America and has invaded Europe, causing substantial economic damage [17]. Feeding larvae produce galleries in leaves and green and ripe fruits, causing considerable damage and ultimately yield losses [18].

Natural enemies are used worldwide for the management of *T. absoluta* in tomato open fields and greenhouses [19,20]. Among them, mirid predators, such as *Nesidiocoris tenuis* (Reuter) and *Macrolophus pygmaeus* Rambour (Hemiptera: Miridae), and *Trichogramma* egg-parasitoids are the most promising for successful biological control of *T. absoluta* [20,21,22].

In tomato, infestation by *T. absoluta* has been demonstrated to induce the emission of HIPVs [23,24]. In addition, egg deposition by *T. absoluta* seems to induce the release of OIPVs by tomato plants [25]. The utilization of HIPVs emitted by tomato plants has been shown to occur for mirid predators [26] and larval parasitoids [27] as part of their foraging behavior. Although the nature of HIPVs for *T. absoluta* larval feeding has been studied in detail [24], there is limited knowledge on the OIPVs by egg deposition of *T. absoluta* females [25,28]. In addition, the role of these OIPVs in the foraging behavior of egg parasitoids, such as *Trichogramma*, which have great potential as a biocontrol agent for *T. absoluta*, has not been elucidated yet. Recently, Gontijo et al. [28] reported behavioral studies for *T. achaeae* to OIPVs and HIPVs emitted by tomato plants.

In the present study, we aimed to address in detail the nature of OIPVs emitted by tomato plants and perceived by the antenna of *Trichogramma* parasitoids. Specifically, we identified electrophysiologically active compounds in the headspace extracts of tomato plants with *T. absoluta* eggs and conducted behavioral tests using a Y-tube olfactometer to investigate the choices of naïve *Trichogramma* parasitoids on OIPVs from tomato plants.

## 2. Materials and Methods

### 2.1. Insects and Plants

The initial population of *T. absoluta* originated from a greenhouse tomato culture at the premises of Benaki Phytopathological Institute (Kifisia, Attica, Greece). Rearing was maintained on tomato plants (*S. lycopersicon* cv. “Missouri” ASGROW^®^), under controlled environmental conditions at 25 ± 1 °C, RH 65 ± 5%, and a photoperiod of 16:8 (L:D). Tomato plants (3–5-week-old plants) were provided to larvae three times a week until pupation. Two *Trichogramma* species were used in the current study, *T. achaeae* Nagaraja and Nagarkatti and *T. cordubense* Vargas and Cabello, with the former obtained from a local commercial company (Anthesis Ltd., Kifisia, GR) and the later from Dr Annette Herz (Julius Kuhn Institute Darmstadt, Germany). Both parasitoid species were reared on sterile *Ephestia kuehniella* eggs obtained from a laboratory colony maintained on semolina flour [29].

### 2.2. Y-Tube Olfactometer Behavioral Experiments

Olfactometer behavioral bioassays were carried out to test the response of the two *Trichogramma* species to the volatile compounds of the tomato. The responses were assessed in a glass Y-tube olfactometer with a 1-cm internal diameter, 10-cm main arm length, and side arms 8 cm long. The olfactometer was lined underneath with filter paper and lightened from above with three 18-W cool fluorescent tubes providing uniform lighting. Air was pumped (Dymax 5, Charles Austen Pumps Ltd., West Byfleet, UK) through an active charcoal filter and re-humidified by passing it through a bottle with tap water before being directed into the two arms of the olfactometer. The air flow rate was adjusted to 30 mL/min. Female parasitoids of both species were subjected to the following tests: (i) Tomato plant with *T. absoluta* eggs 24 h after oviposition versus clean air; (ii) tomato plant with *T. absoluta* eggs 48 h after oviposition versus clean air; and (iii) tomato plant with *T. absoluta* eggs 72 h after oviposition versus clean air. *Trichogramma* parasitoids were released individually at the entrance of the main arm and left for 5 min to make a choice. A single potted tomato plant was placed inside a 10-L glass chamber, which was connected to an arm of the olfactometer. The pot of the plant was covered with aluminum. In all bioassays, after each run, the olfactometer was rotated by 90° to avoid any directional bias. After five replicates, the olfactometer was thoroughly washed with soap and water and rinsed with acetone before being oven-dried at 120 °C. A choice was recorded when a parasitoid crossed 2 cm within the side arm and stayed there for 15 s. At least 30 replicates were performed for each treatment combination on at least 5 different days.

### 2.3. Oviposition-Induced Volatiles

Oviposition-induced volatiles were provoked by placing a tomato plant at the stage of 4 fully grown leaves into cubic cages (60 × 60 × 60 cm) covered by organdy gauze (BugDorm, Taichung, Taiwan) with approximately 30 *T. absoluta* females and removed 24 h later. The cages with tomato plants and *T. absoluta* females were kept under the same experimental conditions as described above. On average, each plant had 12 *T. absoluta* eggs on its leaves. Tomato plants with eggs 24, 48, and 72 h after oviposition were used for the collection of volatiles. Clean tomato plants were used as controls and were maintained in similar experimental conditions but in a separate room to avoid any plant–plant interaction [30]. Five plants were used in each treatment.

### 2.4. Headspace Collection and Identification

The collection of volatiles was done as described by Anastasaki et al. [31]. A single potted tomato plant was placed in a glass container (10 L), with the pot and soil covered with aluminum foil to prevent interaction with VOCs from the soil and roots, and was left for 30 min for acclimatization prior to volatile collection. Purified air, through an activated charcoal filter (10-cm length x 1.5-cm id), was passed through the glass container. Plant volatiles were drawn by a vacuum pump (Dymax 5, Charles Austen Pumps Ltd., West Byfleet, UK) at a rate of 360 mL/min onto a Teflon-made trap (5-cm length x 4-mm id) containing 75 mg Porapak Q (80/100 mesh, Supelco, Bellefonte, PA, USA) tapped with a 2-mm glass wool and 3-mm Teflon tubes in each end. Prior to the analysis, traps were sequentially washed with 1 mL of methanol, diethyl ether, and n-pentane (Fisher Chemicals, Bishop, UK) and blown dry with N_2_. The collection of headspace volatiles was done for 6 h. Immediately after volatile collection, traps were extracted with 500 μL of n-pentane. Sample volumes were reduced to 100 μL and stored in a freezer (at −20 °C) in a sealed vial with a conical inserter until use.

### 2.5. Gas Chromatography-Flame-Ionization-Electroantennographic Detection (GC-FID-EAD)

Plant headspace extracts were subjected to coupled gas chromatography-electroantennogram detection. The system consisted of a Thermo Scientific TRACE 1300 Series GC chromatograph (Milan, Italy) equipped with a flame ionization detector (FID) and coupled to an electroantennogram recording Syntec IDAC-2 (Syntec, Kirchzarten, Germany). Two microliters of each extract were injected manually in the splitless mode. A TG-1 ms capillary column (30 m, 0.25 mm i.d., 0.25-μm film thickness) with helium as the carrier gas at 1 mL/min was used for the analysis of the samples. The column temperature was initially kept for 1 min at 50 °C, then gradually increased to 170 °C at a rate of 3 °C/min, and then at a rate 10 °C/min to 250 °C. The injector and detector temperatures were set at 220 and 250 °C, respectively. The column effluent was mixed with 30 mL/min make-up helium and then spilt at a ratio 1:1 into two branches,—one leading to the FID and the other one through a heated (250 °C) transfer line (Syntec, Kirchzarten, Germany) leading to a glass tube—mixed with a charcoal-filtered, humidified, and constant airstream directed to the antenna controlled by a stimulus controller (CS 55, Syntec, Kirchzarten, Germany). Glass capillaries filled with 0.1 M KCl were used as electrodes. Silver wires were used for electrical contact. The base of the abdomen of a female wasp was mounted on the reference electrode and the top of the antennae placed in the recording electrode. Electrodes were put in the appropriate holder and connected to the probe (Syntec, Kirchzarten, Germany). The mounted insect was placed 0.5 cm from the end of the glass tube. Five successful GC–EAD recordings with different female antennae were performed. Data acquisition was analyzed with GcEad 32software (Syntec, Kirchzarten, Germany). For the quantification, the external standard method was performed (IOFI, 2011). The peak areas of analytes were quantified through external standard calibration curves with standard synthetic compounds. Calibrations curves relating peak areas and concentrations were constructed and expressed in units of μg/h. In the cases where no standard samples were available, the quantification was done with standards of a similar molecular structure. Unknown compounds were quantified in terms of n-alkane with similar retention times. Peak areas for each compound were integrated using Chromeleon 7 software version 7.2.1.5537 (Thermo Scientific, Milan, Italy).

The identification of volatiles from headspace extracts was performed in terms of gas chromatography-mass spectrometry (GC-MS). One microliter of the extract was used for the analysis. It was injected in a Varian CP-3800 GC, with a 1079 injector coupled with a 1200-L quardpupole mass spectrometer. Separation of the analytes was performed with a Varian VF5ms capillary column (30 m, 0.25 mm i.d, 0.25-μm film thickness). The splitless mode was set for 0.75 min. Then, the injector split ratio was set at 80:1. At 5 min, the split ratio was set at 70:1. The flow rate of the carrier gas, helium, was 1 mL/min. The oven temperature was maintained at 40 °C for 1 min, increased at a rate of 1.2 °C/min to 65 °C, and at a rate at 3 °C/min to 180 °C. The column was heated at a rate of 15 °C/min to the final temperature of 250 °C. The mass spectrometer was operated in electron ionization mode (EI) at an ion energy of −70 eV, filament current of 50 μA, and source temperature of 200 °C. Data acquisition was performed in full scan (MS) with the scanning range 40–300 amu. Tentative identification was achieved by comparing the elution order, mass spectra from Adams 2007, NIST 2005, and Wiley 275 mass spectra libraries, and the literature data [32]. We also used retention indices (RI) of a series of n-alkane (C_8_–C_20_). Wherever possible, the retention time and mass spectra were compared with commercial standards.

### 2.6. Statistical Analysis

Chi square test was used for the analysis of the olfactometer data using SPSS [33].

Volatile compounds, measured as peak area and quantified using the external calibration curve, were tested for significant differences between treatments with the non-parametric Kruskal–Wallis H test. The resulting data were log-transformed and processed by projections to latent structures-discriminant analysis (PLS-DA) using SIMCA14.1 software (Umetrics, Umeå, Sweden). The Pareto scaling method was applied to the dataset before PLS-DA processing.

## 3. Results

### 3.1. Response to Olfactometer

**Figure 1:**
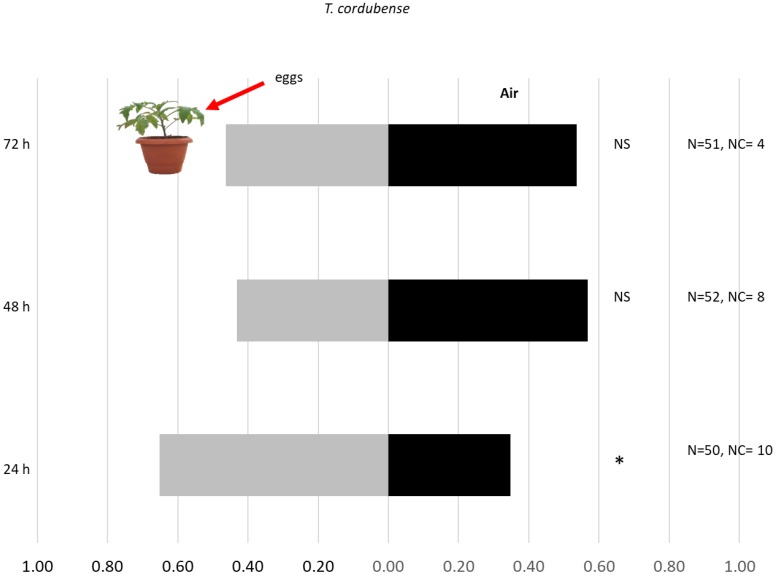
Response of *Trichogramma cordubense* females towards OIPVs from tomato plants induced by *T. absoluta* at 24, 48, and 72 h after oviposition. N, the number of replicates, NC, number of individuals with no choice, NS, not significant, * *p* < 0.05.

**Figure 2:**
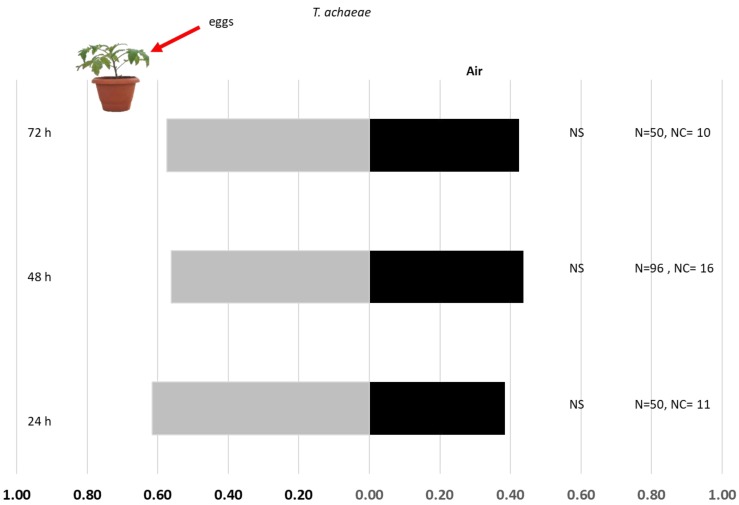
Response of *T. achaeae* females towards OIPVs from tomato plants induced by *T. absoluta* at 24, 48, and 72 h after oviposition. N, the number of replicates, NC, number of individuals with no choice, NS, not significant.

Headspace volatiles from tomato plants with *T. absoluta* eggs 24 h after oviposition were attractive to the egg parasitoid *T. cordubense* (χ^2^ = 4.26, df = 1, *p* = 0.039) (Figure 1). Headspace volatiles from tomato plants with *T. absoluta* eggs 48 and 72 h post-oviposition were not found to be attractive for *T. cordubense* females (χ^2^ = 1.46, df = 1, *p* = 0.23; df = 1, χ^2^ = 0.22, *p* = 0.64) (Figure 1). Although 61.5% of *T. achaeae* females were attracted to the headspace volatiles from tomato plants with *T. absoluta* eggs 24 h after oviposition, this was not statistically significant (χ^2^ = 2.10, df = 1, *p* = 0.15) (Figure 2). *Trichogramma achaeae* females were not attracted by the headspace volatiles of tomato plants with *T. absoluta* eggs 48 and 72 h post-oviposition (χ^2^ = 1.49, df = 1, *p* = 0.22; χ^2^ = 1.19, df = 1, *p* = 0.274) (Figure 2).

### 3.2. Headspace Volatiles

Oviposition by *T. absoluta* induced the emission of a different profile of headspace volatiles by tomato plants compared to tomato plants without eggs of *T. absoluta* (Table 1). *T. absoluta* oviposition significantly enhanced the total emission of VOCs by tomato plants between the different egg treatments (χ^2^ =12.783, df = 3, *p* = 0.005). In total, 68 compounds were identified from the tomato plants, with 9 compounds being isolated only from oviposited tomato plants (Table 1). Major components that were identified in all plant treatments were *β*-phellandrene, 2-*δ*-carene, *α*-phellandrene, and *β*-caryophyllene. In addition, the emission of 19 compounds differed significantly between the control and tomato plants with *T. absoluta* eggs (Table 1).

**Figure 3:**
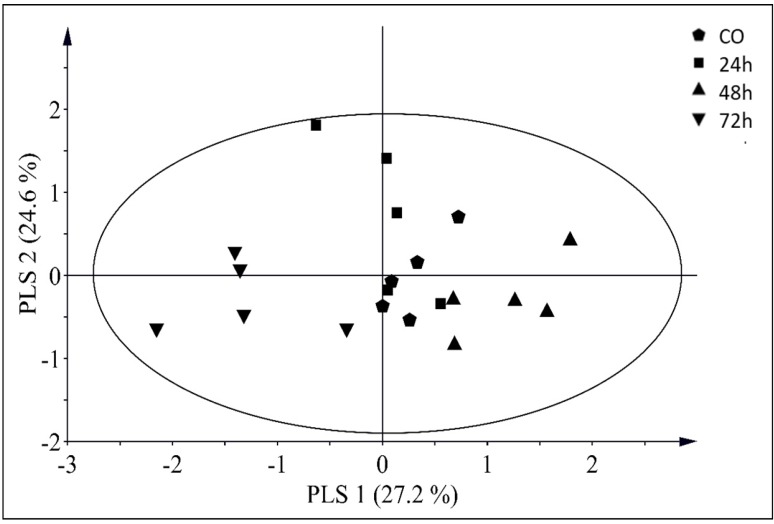
Projection to latent structures discriminant analysis (PLS-DA) score plot of the quantities of volatile compounds emitted from *Tuta absoluta* oviposited plants (24, 48, and 72 h) or control (CO) plants, where the structure of the samples according to the first two PLS components with the explained variance in brackets are visualized. The ellipse defines Hotelling’s T^2^ confidence region (95%).

Projection to latent structures discriminant analysis (PLS-DA) revealed a clear separation between *T. absoluta* egg treatments and control plants (Figure 3). The first two principal components explained 27.2% and 24.6% of the variance, respectively. The PLS-DA analysis identified 28 compounds with a variable importance for the projection (VIP) value higher than 1 (Table 2). A variable with a VIP value close to or greater than 1 can be considered important in a given model. VIP values estimate the importance of each variable (compound) in the projection used in a PLS model and are often used for variable selection. These compounds in decreasing VIP values were: α-phellandrene, 2-δ-carene, *β*-phellandrene, benzyl alcohol, verbenene, α-terpinene, *β*-caryophyllene, *β*-myrcene, *δ*-elemene, nonanal, *α*-pinene, p-cymene, (*E*)-*β*-ocimene, allo-aromadendrene epoxide, *γ*-terpinene, *α*-humulene, germacrene B, (*E*)-isocitral, terpinolene, muurola-4,10 (14)-dien-1b-ol, *β*-elemene, sabinene, unknown 5, p-xylene, terpene 1, hydrocarbon 1, camphor, and unknown 2. In addition, nonanal, p-cymene, and germacrene B contributed the most to the separation of tomato plants with *T. absoluta* eggs 24 h after oviposition.

### 3.3. Identification of EAD Active Compounds

**Figure 4:**
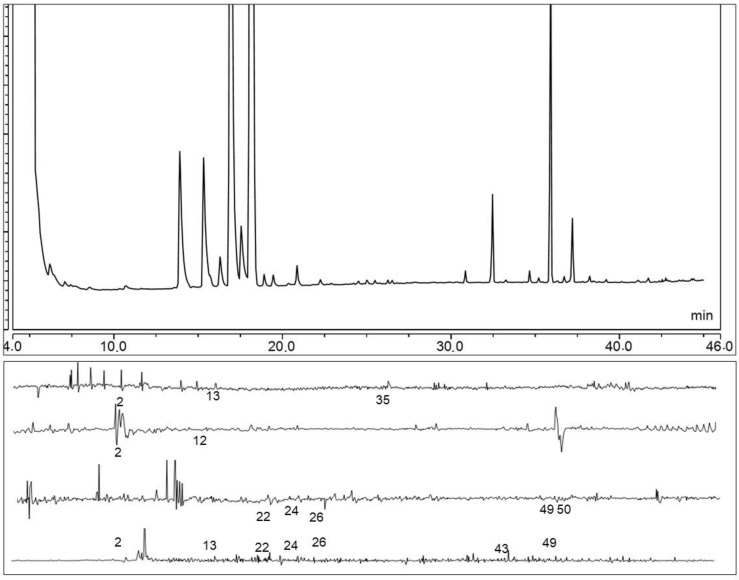
Representative GC-EAD response of female *T. achaea* antenae to volatiles collected from the *T. absoluta* oviposited tomato plant headspace. There are five successful replicates for each extract. For the number interpretation, please refer to Table 1.

Gas chromatography coupled with electro-antennographical detection (GC-EAD) was employed to test the headspace volatiles of oviposited tomato plants. The results showed that parasitoids gave responses to volatiles from tomato plants after the oviposition of *T. absoluta*. Terpenes like *β*-pinene, *β*-myrcene, *γ*-terpinene, *γ*-elemene, and guaidiene-6, 9; aldehydes like nonanal and decanal; and alcohols like 3-(*Z*)-hexen-1-ol were EAD-active compounds (Figure 4). Additionally, unknown compound 5 was found to be EAD active. Parasitoids’ antennae responded to compounds that were relatively small components of these tomato plant extracts. Parasitoids did not respond to the main compounds *β*-phellandrene, 2-*δ*-carene, and *β*-caryophyllene of the tomato volatile blend.

## 4. Discussion

Our study revealed that oviposition-induced volatiles by *T. absoluta* affect the behavior of egg parasitoids. The behavioral response of the parasitoids depends on the species and on the time since oviposition. *Trichogramma cordubense* was attracted to volatiles from tomato plants with *T. absoluta* eggs 24 h after oviposition whereas *T. achaeae* did not discriminate between egg-infested tomato plants. Similarly, Gontijo et al. [28], did not find any attraction of *T. achaea* to tomato plants with eggs of *T. absoluta*. They did find, however, an attraction of *T. achaea* females to the pheromone of *T. absoluta*. It has been shown that egg parasitoids utilize the pheromone of their host as a kairomone to locate patches with hosts’ eggs [29,36]. Nevertheless, a number of studies have shown that OIPVs serve as cues for foraging parasitoids [7,8,37]. Although it was first considered as a plant’s response to wound oviposition [7], later studies have shown that oviposition itself is responsible for the induction of qualitative and quantitative changes in the volatile profile of egg-infested plants [15,25]. Plants definitely benefit by an early activation of defense mechanisms by egg deposition, which enhances their defense before any damage can occur [7,38].

In our GC-EAD experiments, several compounds were found to be detectable by female parasitoids’ antennae. Electrophysiological studies on *Trichogramma* are rare and to our knowledge, no study performing GC-EAD has been conducted. A single study has shown that, using EAG recordings, *T. chilonis* female antennae responded to several compounds belonging to diverse chemical groups, including monoterpenes and the sesquiterpene *β*-caryophyllene [39]. In the current study, *Trichogramma* females responded to OIPVs, such as 3-(*Z*)-hexen-1-ol. Electrophysiological analyses revealed that *Trichogramma* females responded mostly to the minor compounds and they did not, however, respond to the main compounds of *β*-phellandrene, 2-*δ*-carene, and *β*-caryophyllene of the tomato volatile blend. Small qualitative differences are usually more important than obvious quantitative differences in volatiles that affect insect behavior [38] Recently, we showed [31] that *T. absoluta* female antennae can perceive compounds that interfere in oviposition behavior. Compounds, such as *β*-myrcene and 3-(*Z*)-hexen-1-ol, were found to also be detectable by *T. absoluta* females’ antennae. The first one was found to be significantly increased in infested tomato plants while the latter only in infested plants. These compounds seem to have a function in tritrophic interactions. This dual perception by both herbivores and parasitoids confirms that egg deposition produces VOCs that act either as a deterrent for conspecifics or attractant for their natural enemies.

This study confirmed that oviposition by *T. absoluta* induces changes in the volatiles emitted by tomato plants. In the current study, as many as 68 compounds were isolated from *T. absoluta* oviposited tomato plants whereas, in a previous study using another technique for volatile collection, 20 compounds were isolated from the same tomato variety [25]. Here, the 68 compounds were isolated from tomato plants with *T. absoluta* eggs 72 h after oviposition. In a recent study [28], a total of 15 compounds were identified from tomato plants with *T. absoluta* eggs. The profile reported here is similar to other reported data for tomato plants [40,41,42]. The main components were *β*-phellandrene, *2-δ*-carene, *α*-phellandrene, and *β*-caryophyllene. It should be noted that nine compounds were isolated only from tomato plants with *T. absoluta* eggs and they were not detected on clean plants. In addition, the emission of several compounds differed significantly between control and tomato plants with *T. absoluta* eggs due to the higher emission rates from oviposited plants. For instance, (*Z*)-*3*-hexen-1-ol and methyl-salicylate, which are known HIPVs, were isolated only from tomato plants with *T. absoluta* eggs. Gontijo et al. [28] found methyl-salicylate in large amounts from tomato plants with eggs but not (*Z*)-3-hexen-1-ol.

Tomato plants with *T. absoluta* eggs 72 h after oviposition were found to emit a higher number of volatile compounds and also had increased emission of volatiles compared to tomato plants with *T. absoluta* eggs at 24 and 48 h as well as to clean tomato plants. It is known that herbivory enhances the emission of plant volatiles, which is used by natural enemies to locate their hosts [43]. In our conditions, egg hatching occurred within 5 days after oviposition. It is likely that eggs were already not suitable for oviposition and the development of *Trichogramma* larvae. For instance, *Trichogramma* species parasitized more young eggs than older eggs and even when eggs 4 days old were parasitized, no adults finally emerged from them [44]. This could partly explain the absence of an observed attraction of *Trichogramma* females in our study towards tomato plants bearing relatively old *T. absoluta* eggs. Enhanced emission of volatiles by plants as a response to oviposition has been found to be utilized not only by egg parasitoids but also by early larval parasitoids. Larval parasitoids, by distinguishing oviposited plants, have the advantage of locating their hosts at an early developmental stage, which is probably more susceptible to parasitism. Koinobiont parasitoids that attack early larval instars would benefit from being able to identify a plant with eggs close to hatching by saving time and avoiding patches with older host larvae [45].

## 5. Conclusions

Egg parasitoids distinguish oviposition by *T. absoluta* tomato plants and respond to individual compounds identified in these plants based on OIPVs. Understanding the plant–insect interactions and elucidating the behavior of egg parasitoids *Trichogramma* would allow us to manipulate certain interactions to our advantage for proper insect population management with a view to sustainable and biological control of the *T. absoluta* pest in the cultivation of tomato plants.

## Figures and Tables

**Table 1:** Volatile emissions of compounds emitted from *Tuta absoluta* oviposited plants and control plants in μg/h ± SE.

No	RI ^1^	RI_L_^2^	Compound	Identification	Control	Hours after Oviposition			*p* Value
						24 h	48 h	72 h	
1	800	800 ^A^	octane	STD, MS, RI	0.004 ± 0.004	0.003 ± 0.002	nd	0.003 ± 0.003	0.584
2	853	853 ^B^	(*Z*)-3-hexen-1-ol	STD, MS, RI	nd ^3^	nd	nd	0.394 ± 0.381	0.097
3	858	858 ^B^	p-xylene	MS, RI	0.003 ± 0.001	0.008 ± 0.005	0.001 ± 0.001	0.008 ± 0.005	0.404
4	864	864 ^B^	m-xylene	MS, RI	0.004 ± 0.002	0.009 ± 0.004	0.002 ± 0.001	0.003 ± 0.003	0.404
5	887	890 ^B^	o-xylene	MS, RI	nd	0.003 ± 0.002	nd	0.006 ± 0.006	0.171
6	921	924 ^A^	*a-*thujene	MS, RI	0.001 ± 0.000 ^a,5^	0.001 ± 0.000 ^a^	nd ^a^	0.005 ± 0.002 ^b^	0.018
7	932	932 ^A^	*a*-pinene	STD, MS, RI	0.553 ± 0.042	0.541 ± 0.038	0.465 ± 0.024	0.727 ± 0.124	0.128
8	955		Unk 1 ^4^	*m/z*:105, 120, 91	0.002 ± 0.002	0.004 ± 0.002	0.004 ± 0.002	0.005 ± 0.002	0.672
9	958		Unk 2	*m/z*:105, 120, 106, 77	0.005 ± 0.003	0.002 ± 0.001	nd	0.010 ± 0.007	0.195
10	970	970 ^C^	verbenene	MS, RI	0.381 ± 0.007^a^	0.461 ± 0.055 ^a,b^	0.338 ± 0.022 ^a^	0.570 ± 0.052 ^b^	0.020
11	973	974 ^C^	sabinene	STD, MS, RI	0.016 ± 0.005	0.036 ± 0.012	0.011 ± 0.004	0.038 ± 0.013	0.199
12	978	980 ^A^	*β*-pinene	STD, MS, RI	0.001 ± 0.001	0.001 ± 0.001	nd	0.001 ± 0.0000	0.498
13	990	988 ^A^	*β*-myrcene	STD, MS, RI	0.153 ± 0.017	0.181 ± 0.040	0.103 ± 0.014	0.176 ± 0.016	0.091
14	1000	1001 ^A^	2-*δ*-carene	MS, RI	2.731 ± 0.220 ^a,b^	2.435 ± 0.137 ^a^	2.190 ± 0.358 ^a^	3.883 ± 0.643 ^b^	0.032
15	1005	1002 ^A^	*α*-phellandrene	STD, MS, RI	0.462 ± 0.041 ^a,b^	0.446 ± 0.009 ^a^	0.380 ± 0.047 ^a^	0.700 ± 0.086 ^b^	0.010
16	1015	1014 ^A^	*α*-terpinene	STD, MS, RI	0.166 ± 0.021 ^a,b^	0.161 ± 0.020 ^a,b^	0.117 ± 0.027 ^a^	0.237 ± 0.027 ^b^	0.034
17	1024	1020 ^A^	p-cymene	STD, MS, RI	0.041 ± 0.017	0.042 ± 0.015	0.012 ± 0.002	0.025 ± 0.008	0.146
18	1029	1031 ^C^	*β*-phellandrene	MS, RI	7.556 ± 0.358 ^a^	8.333 ± 0.419 ^a,b^	6.995 ± 0.980 ^a^	11.402 ± 0.987 ^b^	0.011
19	1035	1032 ^E^	benzyl alcohol	MS, RI	nd ^a^	nd ^a^	0.002 ± 0.001 ^a,b^	0.089 ± 0.041 ^b^	0.011
20	1038	1037 ^A^	(*Z*)-*β*-ocimene	MS, RI	0.017 ± 0.003	0.020 ± 0.004	0.006 ± 0.002	0.025 ± 0.009	0.060
21	1049	1044 ^A^	(*E*)-*β*-ocimene	STD, MS, RI	0.073 ± 0.004 ^b^	0.048 ± 0.010 ^a^	0.047 ± 0.006 ^a^	0.083 ± 0.017 ^b^	0.047
22	1059	1054 ^A^	*γ*-terpinene	STD, MS, RI	0.025 ± 0.001 ^a^	0.021 ± 0.002 ^a^	0.029 ± 0.006 ^a,b^	0.037 ± 0.003 ^b^	0.035
23	1085	1086 ^A^	terpinolene	STD, MS, RI	0.032 ± 0.004 ^a,b^	0.039 ± 0.010 ^a,b^	0.026 ± 0.004 ^a^	0.049 ± 0.004 ^b^	0.029
24	1108	1108 ^C^	nonanal	STD, MS, RI	0.027 ± 0.015	0.095 ± 0.042	0.036 ± 0.016	0.025 ± 0.009	0.336
25	1115		Terpene 1	*m/z*:93, 136, 121, 91, 79	0.008 ± 0.002 ^a,b^	0.004 ± 0.002 ^a^	0.010 ± 0.001 ^a,b^	0.017 ± 0.003 ^b^	0.011
26	1122	1118 ^A^	cis-p-menth-2-en-1-ol	MS, RI	nd	nd	0.001 ± 0.001	0.002 ± 0.001	0.061
27	1124	1119 ^A^	trans-p-mentha-2,8-dien-1-ol	MS, RI	0.001 ± 0.000	nd	0.001 ± 0.001	0.015 ± 0.014	0.102
28	1133	1133 ^A^	cis-p-mentha-2,8-dien-1-ol	MS, RI	0.002 ± 0.001	0.002 ± 0.001	0.002 ± 0.001	0.006 ± 0.002	0.177
29	1141	1141 ^A^	camphor	STD, MS, RI	0.007 ± 0.005	nd	0.007 ± 0.005	0.001 ± 0.000	0.357
30	1173		Unk 3	*m/z*:109,79,91	0.005 ± 0.004	nd	0.001 ± 0.001	0.007 ± 0.004	0.107
31	1175	1177 ^A^	(*E*)-isocitral	MS, RI	0.004 ± 0.001	0.007 ± 0.004	0.003 ± 0.002	0.019 ± 0.006	0.211
32	1185	1184 ^A^	dill ether	MS, RI	0.008 ± 0.002	0.007 ± 0.005	0.008 ± 0.002	0.014 ± 0.003	0.151
33	1195	1195 ^C^	methyl salicylate	STD, MS, RI	nd^a^	nd^a^	0.002 ± 0.001^a^	0.025 ± 0.009 ^b^	0.001
34	1200	1200 ^A^	dodecane	STD, MS, RI	0.022 ± 0.010	0.039 ± 0.018	0.020 ± 0.007	0.031 ± 0.017	0.902
35	1208	1208 ^C^	decanal	STD, MS, RI	0.014 ± 0.007	0.032 ± 0.013	0.019 ± 0.008	0.015 ± 0.007	0.417
36	1231	1232 ^A^	(Z)-3-hexenyl-2-methyl butanoate	STD, MS, RI	nd ^a^	nd ^a^	0.004 ± 0.002 ^b^	0.003 ± 0.002 ^a,b^	0.017
37	1237	1234 ^A^	ascaridole		0.003 ± 0.001 ^b^	nd ^a^	0.001 ± 0.000 ^a,b^	0.003 ± 0.001 ^b^	0.023
38	1247	1244 ^A^	car-3-en-2-one	MS, RI	0.001 ± 0.001	nd	nd	0.001 ± 0.000	0.095
39	1300	1300 ^A^	tridecane	STD, MS, RI	0.022 ± 0.010	0.011 ± 0.006	0.015 ± 0.008	0.007 ± 0.002	0.478
40	1304		Unk 4	*m/z*:97, 54, 69	0.003 ± 0.001	0.001 ± 0.001	0.001 ± 0.001	0.004 ± 0.002	0.112
41	1333	1335 ^A^	*δ*-elemene	MS, RI	0.132 ± 0.016	0.173 ± 0.027	0.111 ± 0.020	0.195 ± 0.044	0.448
42	1349		Ester 1	*m/z*: 71, 83	0.001 ± 0.001	nd	nd	0.010 ± 0.007	0.100
43	1355		Unk 4	*m/z*:57, 71, 85	0.001 ± 0.001	nd	nd	0.017 ± 0.015	0.100
44	1370		Ester 2	*m/z*:71, 89, 56	0.001 ± 0.001	0.001 ± 0.001	nd	0.002 ± 0.002	0.265
45	1374	1374 ^A^	*α*-copaene	MS, RI	0.013 ± 0.007	0.002 ± 0.002	0.001 ± 0.001	0.010 ± 0.002	0.053
46	1387	1389 ^A^	*β*-elemene	STD, MS, RI	0.016 ± 0.002	0.034 ± 0.012	0.014 ± 0.003	0.038 ± 0.012	0.126
47	1400	1400 ^A^	tetradecane	STD, MS, RI	0.059 ± 0.028	0.061 ± 0.028	0.034 ± 0.017	0.033 ± 0.010	0.763
48	1417	1417 ^A^	*β*-caryophyllene	STD, MS, RI	0.367 ± 0.028	0.341 ± 0.033	0.263 ± 0.045	0.517 ± 0.115	0.164
49	1427	1432 ^D^	*γ*-elemene	MS, RI	0.005 ± 0.002	0.002 ± 0.002	0.002 ± 0.000	0.007 ± 0.002	0.056
50	1439	1442 ^A^	guaidiene-6,9	MS, RI	0.010 ± 0.001	0.020 ± 0.006	0.008 ± 0.002	0.014 ± 0.004	0.179
51	1447	1448 ^A^	muurola-3,5-diene	MS, RI	0.005 ± 0.002	0.001 ± 0.001	nd	0.003 ± 0.001	0.085
52	1459	1459 ^D^	*α*-humulene	STD, MS, RI	0.077 ± 0.008	0.075 ± 0.006	0.050 ± 0.008	0.103 ± 0.023	0.114
53	1481	1484 ^A^	germacrene D	MS, RI	0.011 ± 0.000	0.009 ± 0.003	0.010 ± 0.003	0.016 ± 0.003	0.281
54	1495	1500 ^A^	*α*-muurolene	MS, RI	0.006 ± 0.002 ^b^	0.001 ± 0.001 ^a^	0.001 ± 0.001 ^a^	0.005 ± 0.001 ^b^	0.008
55	1500	1500 ^A^	pentadecane	STD, MS, RI	0.011 ± 0.006	0.018 ± 0.006	0.009 ± 0.003	0.014 ± 0.005	0.207
56	1504	1508 ^Α^	germacrene Α	MS, RI	0.001 ± 0.001 ^b^	nd ^a^	nd ^a^	0.004 ± 0.003 ^b^	0.020
57	1524		Terpene 2	*m/z*:121, 93, 91, 105, 161	0.001 ± 0.000 ^a^	0.001 ± 0.000 ^a^	nd ^a^	0.003 ± 0.001 ^b^	0.003
58	1552		Unk 5	*m/z*:55, 83, 69	0.005 ± 0.002 ^b^	0.001 ± 0.001 ^a^	0.001 ± 0.000^a^	0.005 ± 0.002 ^b^	0.007
59	1557	1559 ^A^	germacrene B	MS, RI	0.008 ± 0.002 ^b^	0.016 ± 0.005 ^b^	0.002 ± 0.001^a^	0.008 ± 0.002 ^b^	0.018
60	1562	1561 ^A^	nerolidol	STD, MS, RI	0.007 ± 0.003 ^c^	0.001 ± 0.001 ^b^	nd ^a,b^	0.008 ± 0.002 ^c^	0.002
61	1574	1573 ^C^	(*Ε-Ε*)-TMTT	MS, RI	0.016 ± 0.004	0.014 ± 0.007	0.003 ± 0.001	0.004 ± 0.001	0.152
62	1581	1582 ^A^	caryophyllene oxide	STD, MS, RI	0.008 ± 0.002	0.003 ± 0.002	0.006 ± 0.001	0.013 ± 0.005	0.229
63	1598		Terpene 3	*m/z*:93, 80, 121, 149	nd ^a^	nd ^a^	nd ^a^	0.017 ± 0.015 ^b^	0.003
64	1600	1600 ^A^	hexadecane	STD, MS, RI	0.278 ± 0.145	0.136 ± 0.055	0.117 ± 0.056	0.146 ± 0.068	0.831
65	1608	1608 ^A^	Humulene epoxide II	MS, RI	0.004 ± 0.004	nd	nd	0.044 ± 0.031	0.222
66	1621		Terpene 4	*m/z*: 81, 161, 105, 119, 93	nd	nd	0.016 ± 0.011	0.080 ± 0.068	0.195
67	1630	1630 ^A^	muurola-4,10 (14)-dien-1b-ol	MS, RI	0.004 ± 0.001	0.002 ± 0.001	nd	0.011 ± 0.006	0.078
68	1641	1639 ^A^	Allo-aromadendrene epoxide	MS, RI	nd	nd	nd	0.081 ± 0.080	0.222
			Total		13.40 ± 0.43 ^a^	13.90 ± 0.41 ^a^	11.51 ± 1.35 ^a^	20.08 ± 1.91 ^b^	0.005

^1^ Retention Index relative to C_8_–C_20_ n-alkanes on a VF5ms column. ^2^ Retention Index obtained from [32] ^A^, [34] ^B^, [31] ^C^, [25] ^D^, [35] ^E^. ^3^ not detected. ^4^ Unknown. ^5^ Means followed by different letter (a, b, c) within a row, are significantly differ based on the Kruskal–Wallis test *(p* = 0.05).

**Table 2:** Values of variable importance to the projection (VIP) of volatiles.

No.	Compound	VIP Value
1	α-phellandrene	1.97
2	2-δ-carene	1.92
3	*β*-phellandrene	1.88
4	benzyl alcohol	1.84
5	verbenene	1.75
6	α-terpinene	1.70
7	*β-*caryophyllene	1.54
8	*β*-myrcene	1.52
9	*δ*-elemene	1.42
10	nonanal	1.40
11	*α*-pinene	1.39
12	p-cymene	1.29
13	(*E*)-*β*-ocimene	1.28
14	allo-aromadendrene epoxide	1.26
15	*γ*-terpinene	1.24
16	*α-*humulene	1.23
17	germacrene B	1.22
18	*(E)*-isocitral	1.18
19	terpinolene	1.14
20	muurola-4,10 (14)-dien-1b-ol	1.14
21	*β*-elemene	1.13
22	sabinene	1.13
23	unknown 5	1.10
24	p-xylene	1.09
25	terpene 1	1.07
26	hydrocarbon 1	1.06
27	camphor	1.03
28	unknown 2	1.00

## References

[1] Dicke M., Baldwin I.T. (2010). The evolutionary context for herbivore-induced plant volatiles: Beyond the “cry for help”. Trends Plant Sci..

[2] Turlings T.C.J., Tumlinson J.H., Lewis W.J. (1990). Exploitation of Herbivore-Induced Plant Odors by Host-Seeking Parasitic Wasps. Science.

[3] Howe G.A., Jander G. (2008). Plant Immunity to Insect Herbivores. Annu. Rev. Plant Biol..

[4] Heil M. (2008). Indirect defence via tritrophic interactions. New Phytol..

[5] Agrawal A.A. (1999). Induced plant defense: Evolution of induction and adaptive phenotypic plasticity. Inducible Plant Defenses Against Pathogens and Herbivores: Biochemistry, Ecology, and Agriculture.

[6] Agrawal A.A. (2011). Current trends in the evolutionary ecology of plant defence. Funct. Ecol..

[7] Hilker M., Fatouros N.E. (2015). Plant responses to insect egg deposition. Annu. Rev. Entomol..

[8] Colazza S., McElfresh J.S., Millar J.G. (2004). Identification of volatile synomones, induced by Nezara viridula feeding and oviposition on bean spp., that attract the egg parasitoid: *Trissolcus basalis*. J. Chem. Ecol..

[9] Colazza S., Fucarino A., Peri E., Salerno G., Conti E., Bin F. (2004). Insect oviposition induces volatile emission in herbaceous plants that attracts egg parasitoids. J. Exp. Biol..

[10] Fatouros N.E., Bukovinszkine’Kiss G., Dicke M., Hilker M. (2007). The response specificity of Trichogramma egg parasitoids towards infochemicals during host location. J. Insect Behav..

[11] Salerno G., De Santis F., Iacovone A., Bin F., Conti E. (2013). Short-range cues mediate parasitoid searching behavior on maize: The role of oviposition-induced plant synomones. Biol. Control.

[12] Fatouros N.E., Cusumano A., Danchin E.G.J., Colazza S. (2016). Prospects of herbivore egg-killing plant defenses for sustainable crop protection. Ecol. Evol..

[13] Ponzio C., Cascone P., Cusumano A., Weldegergis B.T., Fatouros N.E., Guerrieri E., Dicke M., Gols R. (2016). Volatile-mediated foraging behaviour of three parasitoid species under conditions of dual insect herbivore attack. Anim. Behav..

[14] Cusumano A., Weldegergis B.T., Colazza S., Dicke M., Fatouros N.E. (2015). Attraction of egg-killing parasitoids toward induced plant volatiles in a multi-herbivore context. Oecologia.

[15] Tamiru A., Bruce T.J.A., Woodcock C.M., Caulfield J.C., Midega C.A.O., Ogol C.K.P.O., Mayon P., Birkett M.A., Pickett J.A., Khan Z.R. (2011). Maize landraces recruit egg and larval parasitoids in response to egg deposition by a herbivore. Ecol. Lett..

[16] Frati F., Cusumano A., Conti E., Colazza S., Peri E., Guarino S., Martorana L., Romani R., Salerno G. (2017). Foraging behaviour of an egg parasitoid exploiting plant volatiles induced by pentatomids: The role of adaxial and abaxial leaf surfaces. PeerJ.

[17] Biondi A., Guedes R.N.C., Wan F.-H., Desneux N. (2018). Ecology, Worldwide Spread, and Management of the Invasive South American Tomato Pinworm, *Tuta absoluta*: Past, Present, and Future. Annu. Rev. Entomol..

[18] Desneux N., Wajnberg E., Wyckhuys K.A.G., Burgio G., Arpaia S., Narváez-Vasquez C.A., González-Cabrera J., Ruescas D.C., Tabone E., Frandon J. (2010). Biological invasion of European tomato crops by *Tuta absoluta*: Ecology, geographic expansion and prospects for biological control. J. Pest Sci..

[19] Zappalà L., Biondi A., Alma A., Al-Jboory I.J., Arnò J., Bayram A., Chailleux A., El-Arnaouty A., Gerling D., Guenaoui Y. (2013). Natural enemies of the South American moth, *Tuta absoluta*, in Europe, North Africa and Middle East, and their potential use in pest control strategies. J. Pest Sci..

[20] Urbaneja A., González-Cabrera J., Arnó J., Gabarra R. (2012). Prospects for the biological control of *Tuta absoluta* in tomatoes of the Mediterranean basin. Pest Manag. Sci..

[21] Oliveira L., Durão A.C., Fontes J., Roja I.S., Tavares J. (2017). Potential of *Trichogramma achaeae* (Hymenoptera: Trichogrammatidae) in Biological Control of *Tuta absoluta* (Lepidoptera: Gelechiidae) in Azorean Greenhouse Tomato Crops. J. Econ. Entomol..

[22] Cascone P., Carpenito S., Slotsbo S., Iodice L., Sørensen J.G., Holmstrup M., Guerrieri E. (2015). Improving the efficiency of Trichogramma achaeae to control *Tuta absoluta*. BioControl.

[23] De Backer L., Megido R.C., Fauconnier M.L., Brostaux Y., Francis F., Verheggen F. (2015). *Tuta absoluta*-induced plant volatiles: Attractiveness towards the generalist predator *Macrolophus pygmaeus*. Arthropod. Plant. Interact..

[24] Silva D.B., Weldegergis B.T., Van Loon J.J.A., Bueno V.H.P., Van Loon J.J.A., Bueno V.H.P. (2017). Qualitative and Quantitative Differences in Herbivore-Induced Plant Volatile Blends from Tomato Plants Infested by Either *Tuta absoluta* or Bemisia tabaci. J. Chem. Ecol..

[25] Anastasaki E., Balayannis G., Papanikolaou N.E., Michaelakis A.N., Milonas P.G. (2015). Oviposition induced volatiles in tomato plants. Phytochem. Lett..

[26] Silva D.B., Bueno V.H.P., Van Loon J.J.A., Peñaflor M.F.G.V., Bento J.M.S., Van Lenteren J.C. (2018). Attraction of Three Mirid Predators to Tomato Infested by Both the Tomato Leaf Mining Moth *Tuta absoluta* and the Whitefly Bemisia Tab. J. Chem. Ecol..

[27] Bodino N., Ferracini C., Tavella L. (2016). Is host selection influenced by natal and adult experience in the parasitoid *Necremnus tutae* (Hymenoptera: Eulophidae)?. Anim. Behav..

[28] Gontijo L., Cascone P., Giorgini M., Michelozzi M., Rodrigues H.S., Spiezia G., Iodice L., Guerrieri E. (2019). Relative importance of host and plant semiochemicals in the foraging behavior of *Trichogramma achaeae*, an egg parasitoid of *Tuta absoluta*. J. Pest Sci..

[29] Milonas P.G., Martinou A.F., Kontodimas D.C., Karamaouna F., Konstantopoulou M.A. (2009). Attraction of different Trichogramma species to *Prays oleae* sex pheromone. Ann. Entomol. Soc. Am..

[30] Zakir A., Bengtsson M., Sadek M.M., Hansson B.S., Witzgall P., Anderson P. (2013). Specific response to herbivore-induced de novo synthesized plant volatiles provides reliable information for host plant selection in a moth. J. Exp. Biol..

[31] Anastasaki E., Drizou F., Milonas P.G. (2018). Electrophysiological and Oviposition Responses of *Tuta absoluta* Females to Herbivore-Induced Volatiles in Tomato Plants. J. Chem. Ecol..

[32] Adams R.P. (2007). Identification of Essential Oil Components by Gas Chromatography/Mass Spectrometry.

[33] Agresti A. (2013). Categorical Data Analysis.

[34] Song C., Lai W.C., Reddy K.M., Wei B. (2003). Temperature-programmed retention indices for GC and GC-MS of hydrocarbon fuels and simulated distillation GC of heavy oils. Analytical Advances for Hydrocarbon Research.

[35] Maselou D.A., Anastasaki E., Milonas P.G. (2019). The role of host plants, alternative food resources and herbivore induced volatiles in choice behavior of an omnivorous predator. Front. Ecol. Evol..

[36] Fatouros N.E., Dicke M., Mumm R., Meiners T., Hilker M. (2008). Foraging behavior of egg parasitoids exploiting chemical information. Behav. Ecol..

[37] Tamiru A., Bruce T.J.A., Midega C.A.O., Woodcock C.M., Birkett M.A., Pickett J.A., Khan Z.R. (2012). Oviposition Induced Volatile Emissions from African Smallholder Farmers’ Maize Varieties. J. Chem. Ecol..

[38] Bruce T.J.A., Midega C.A.O., Birkett M.A., Pickett J.A., Khan Z.R. (2009). Is quality more important than quantity? Insect behavioural responses to changes in a volatile blend after stemborer oviposition on an African grass. Biol. Lett..

[39] Sen A., Raina R., Joseph M., Tungikar V.B. (2005). Response of Trichogramma chilonis to infochemicals: An SEM and electrophysiological investigation. BioControl.

[40] Ángeles López Y.I., Martínez-Gallardo N.A., Ramírez-Romero R., López M.G., Sánchez-Hernández C., Délano-Frier J.P. (2012). Cross-Kingdom Effects of Plant-Plant Signaling via Volatile Organic Compounds Emitted by Tomato (*Solanum lycopersicum*) Plants Infested by the Greenhouse Whitefly (*Trialeurodes vaporariorum*). J. Chem. Ecol..

[41] Buttery R.G., Ling L.C., Light D.M. (1987). Tomato leaf volatile aroma components. J. Agric. Food Chem.

[42] Kant M.R., Ament K., Sabelis M.W., Haring M.A., Schuurink R.C. (2004). Differential timing of spider mite-induced direct and indirect defenses in tomato plants. Plant Physiol..

[43] Dicke M. (2009). Behavioural and community ecology of plants that cry for help. Plant. Cell Environ..

[44] Du W.M., Xu J., Hou Y.Y., Lin Y., Zang L.S., Yang X., Zhang J.J., Ruan C.C., Desneux N. (2018). *Trichogramma parasitoids* can distinguish between fertilized and unfertilized host eggs. J. Pest Sci..

[45] Fatouros N.E., Lucas-Barbosa D., Weldegergis B.T., Pashalidou F.G., van Loon J.J.A., Dicke M., Harvey J.A., Gols R., Huigens M.E. (2012). Plant volatiles induced by herbivore egg deposition affect insects of different trophic levels. PLoS ONE.

